# ATN system and disease‐modifying treatment eligibility in a hospital‐based cohort

**DOI:** 10.1002/alz.086761

**Published:** 2025-01-09

**Authors:** Giordano Cecchetti, Elisa Canu, Giulia Rugarli, Federico Coraglia, Silvia Basaia, Sonia Francesca Calloni, Paolo Q. Vezzulli, Edoardo G. Spinelli, Roberto Santangelo, Francesca Caso, Andrea Falini, Giuseppe Magnani, Massimo Filippi, Federica Agosta

**Affiliations:** ^1^ IRCCS San Raffaele Scientific Institute, Milan Italy; ^2^ Vita‐Salute San Raffaele University, Milan Italy

## Abstract

**Background:**

This study aims at applying the AT(N) classification to a cohort of patients with Alzheimer’s disease (AD) and related disorders, and to investigate how many cases would be eligible for the emerging disease‐modifying treatments.

**Method:**

We conducted a retrospective evaluation of 429 patients referred to the Memory Center of IRCCS San Raffaele Hospital in Milan. Patients underwent clinical/neuropsychological assessments, lumbar puncture, structural brain imaging, and positron emission tomography (FDG‐PET). Patients were stratified according to AT(N) classification, group comparisons were performed and the number of eligible cases for anti‐β amyloid monoclonal antibodies was calculated.

**Result:**

Sociodemographic and clinical features were similar across groups. Although the clinical presentation was similar, the A+T+N+ group showed more severe cognitive impairment in memory, language, attention, executive, and visuospatial functions compared to other AT(N) groups. Notably, T+ patients demonstrated greater memory complaints compared to T‐ cases. FDG‐PET outperformed MRI and CT in distinguishing A+ from A‐ patients. Although the 60.8% of the observed cases were A+, only the 17.2% were eligible for amyloid‐targeting treatments.

**Conclusion:**

The AT(N) classification is applicable in a real‐world clinical setting. The classification system provided insights into clinical management and treatment strategies. Low cognitive performance and specific regional FDG‐PET hypometabolism at diagnosis are highly suggestive for A+T+ or A‐T+ profiles. This work provides also a realistic picture of the proportion of AD patients eligible for disease modifying treatments emphasizing the need for early detection.

**Funding**: Foundation Research on Alzheimer Disease. Next Generation EU/National Recovery and Resilience Plan, Investment PE8‐Project Age‐It.

